# SOP for pathway inference in Integrated Microbial Genomes (IMG)

**DOI:** 10.4056/sigs.1193182

**Published:** 2011-12-22

**Authors:** Iain Anderson, Amy Chen, Victor Markowitz, Nikos Kyrpides, Natalia Ivanova

**Affiliations:** 1 DOE Joint Genome Institute, Walnut Creek, CA, USA

## Abstract

One of the most important aspects of genomic analysis is the prediction of which pathways, both metabolic and non-metabolic, are present in an organism. In IMG, this is carried out by the assignment of IMG terms, which are organized into IMG pathways. Based on manual and automatic assignment of IMG terms, the presence or absence of IMG pathways is automatically inferred. The three categories of pathway assertion are asserted (likely present), not asserted (likely absent), and unknown. In the unknown category, at least one term necessary for the pathway is missing, but an ortholog in another organism has the corresponding term assigned to it. Automatic pathway inference is an important initial step in genome analysis.

## Introduction

The assignment of genes to pathways in IMG begins with the assignment of IMG terms. IMG terms are a set of functional assignments curated by members of the Genome Biology Program (GBP) at the Joint Genome Institute (JGI). There are three types of IMG terms: *Gene Product*, *Modified Protein*, and *Protein Complex*. *Gene Product* terms are assigned directly to genes. *Modified Protein* refers to a covalently or non-covalently modified protein, for example a phosphorylated protein or a protein that binds a cofactor. *Protein Complex* terms are associations of two or more IMG terms, where the components of the complex can be any of the three types of IMG terms. This multi-level organization of IMG terms is designed to disambiguate association between reactions and genes in cases of complex catalysts, such as multi-subunit enzymes, which may require multiple cofactors and undergo a multi-step maturation process. As a result, the information about all genes and pathways required for activity of the catalysts is explicitly recorded in IMG, which enables more precise automated inference of the presence of individual reactions and entire pathways. More information about the rationale for IMG terms can be found in the *Using IMG* section of the IMG website [1].

**Figure 1:**
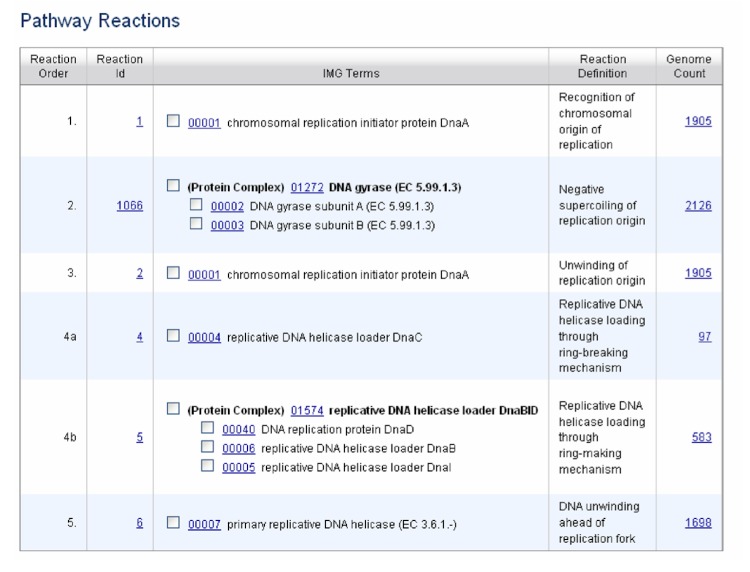
List of reactions and terms involved in the pathway “Bacterial replication initiation” as displayed on the IMG Pathway Details page.

**Figure 2:**
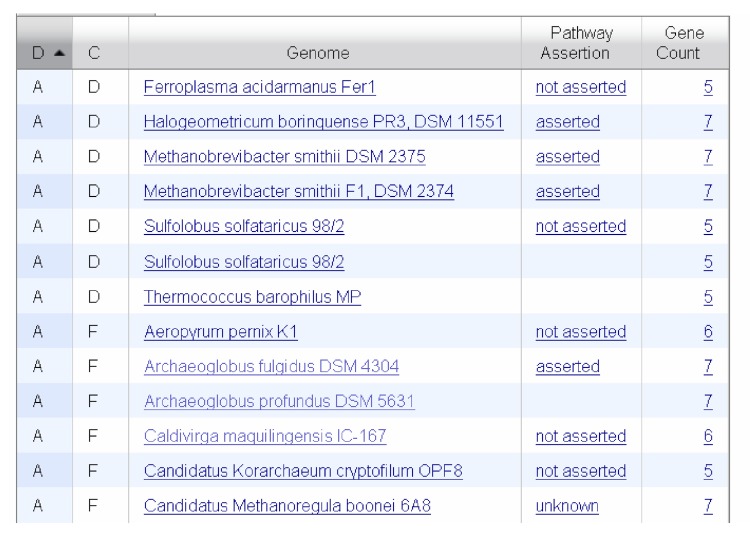
Partial list of genomes from a Pathway Details page showing the number of genes associated with the pathway and the pathway assertion status.

IMG terms participate as catalysts in IMG reactions, which make up IMG pathways. Some IMG reactions are traditional biochemical reactions corresponding to conversion of small molecules with known stoichiometry, while others describe chemical conversions of unknown or undefined stoichiometry, transport of small molecules or macromolecules, protein-protein interactions or other interactions between other macromolecules, such as conformational changes (see BioPax documentation for detailed classification of reactions, [2]). Figure 1 shows the top section of a Pathway Details page with the list of reactions and the terms that act as catalysts for these reactions. The example shown is a non-metabolic pathway, which consists of protein-protein and protein-DNA interactions, as well as topological changes of DNA, as described in the reaction definitions. For a metabolic pathway, the reaction definition would be a biochemical reaction. The bottom section of a Pathway Details page shows a list of organisms, how many genes they have that belong to the pathway, and the pathway assertion status (Figure 2).

With the huge number of genomes available, it would be too large a task to go through each genome individually to determine which pathways are present, so the process must be automated. Automated pathway inference has several goals. The first is to predict which pathways are found in a given organism. If physiological studies of the organism have been carried out, then the experimental results can be compared with the automated pathway inference results. Two other goals of pathway inference are 1) to identify errors in the assignment of IMG terms to genes, and 2) to identify “missing” genes. If a pathway is known to be present in an organism but it is not asserted as being present, then one possibility is that a gene should have a term from this pathway assigned to it, but it was not assigned during the annotation process. If a search for a gene that should have this term assigned is successful, the term can then be assigned and the gap filled. If the search is not successful, then this gene function is *missing*, meaning that it can not be found based on similarity to genes that are known to have this activity. In this case, it is possible that a new protein family will be found to have this activity. Another goal of pathway inference is to find new physiological functions for an organism. Genes for a pathway that was thought to be absent may be found in the genome. For example, *Ferroglobus placidus* is known to perform partial denitrification by reducing nitrate to nitrous oxide, but dinitrogen production was not detected [3]. However, in the genome there is a gene with a strong similarity to nitrous oxide reductases, suggesting that under some conditions this organism may produce dinitrogen.

## Procedure

The first step of automated pathway inference in IMG is to check if catalysts for all reactions of a pathway are present in the organism. In some pathways there are alternative catalysts for certain reactions or alternative reactions that differ in their coenzyme specificity (for instance, NADH or NADPH). In the example in Figure 1 there are two alternatives at step 4, helicase loading through ring-breaking or ring-making mechanism. Both reactions result in assembly of a hexameric ring of replicative DNA helicase with DNA bound in its central channel. However, the mechanism of assembly is different in different bacteria and requires assistance of different proteins called helicase loaders [4]. Only one of these alternatives needs to be present for the pathway to be asserted. If a protein complex carries out a step in the pathway, all components of the complex must be present. For example in Figure 1, step 2 requires subunit A and subunit B of DNA gyrase.

**Figure 3:**
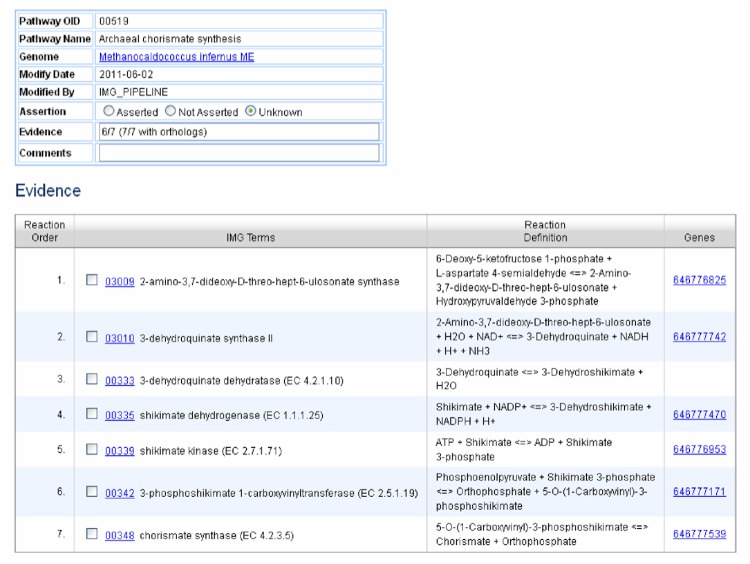
Pathway assertion details page for the pathway Archaeal chorismate synthesis in the organism *Methanocaldococcus infernus*.

If there is at least one term missing from the pathway, there are two possibilities for pathway assignment status – unknown, and not asserted. The assignment of these terms depends on whether or not the genome has a gene with an ortholog that has the assigned function. If no ortholog has the missing function, then the pathway is assigned the status of *not asserted*. If an ortholog is present for the missing step, then the pathway status is *unknown*. Figure 3 shows an example of a pathway with status *unknown*. Genes with six out of the seven IMG terms involved in the pathway are present, and for the single missing term, 3-dehydroquinate dehydratase, there is an ortholog with this function. This is shown in the top table in the Evidence entry where *7/7 with orthologs* is written.

## Implementation

Automatic IMG pathway assertion is implemented in a Perl program that interacts with the IMG Oracle database. The program iterates through all IMG pathways and organisms and checks whether the organism has genes associated with IMG terms needed in each reaction of the pathway. Authorized users are able to manually curate the pathway assertion status. If a pathway has been manually curated for an organism, then the program will skip this combination of pathway and organism. Automatic pathway assertion is done three times per year, in sync with each major release of IMG.

## Discussion/remarks

The interpretation of a sequenced genome requires a comparison between experimentally determined physiological data and analysis of the pathways encoded in the genome sequence. Automatic pathway inference is an initial step for analysis of the capabilities of the organism under study. Discrepancies between the automatic predictions and the known biology can serve as starting points for further experimental studies. Automated pathway assertion is currently available in the public version of IMG.
